
JOURNAL INFORMATION
==============================
NLM Title Abbreviation: ScientificWorldJournal
Journal ID: ScientificWorldJournal
NLM Journal ID: 101131163
Journal ID: TSWJ

The Scientific World Journal

ISSN: 2356-6140
EISSN: 1537-744X
Publisher: Wiley

ARTICLE INFORMATION
==============================
PMCID: PMC6084409
PMID: 30147622
DOI: 10.1100/tsw.2001.246
Article ID: 538745
Article version: 1
Subjects: Short Report

The Impact of Ageing on CHITIN Scars in Saccharomyces cerevisiae

Powell Christopher D. 1
Quain David E. 2
Smart Katherine A. 1 *
1 Department of BMS, Oxford Brookes University, Headington, Oxford, UK
2 Bass Brewers Technical Centre, Burton-On-Trent, UK
*Katherine A. Smart: kasmart@brookes.ac.uk
Publication date: 2001
Electronic publication date: 2001 May 1
Volume: 1
First page: 145
Last page: 145
Copyright: Copyright © 2001 Christopher D. Powell et al.
Copyright year: 2001
License: This is an open access article distributed under the Creative Commons Attribution License, which permits unrestricted use, distribution, and reproduction in any medium, provided the original work is properly cited.
License URL: https://creativecommons.org/licenses/by/3.0/

Funding: Biotechnology and Biological Sciences Research Council

==============================
Miami *Nature Biotechnology* Short Reports

*TheScientificWorld* (2001) 1 (S3), 145SR

ISSN 1532-2246; DOI 10.1100/tsw.2001.246

### THE IMPACT OF AGEING ON CHITIN SCARS IN *SACCHAROMYCES CEREVISIAE*

Christopher D. Powell[1], David E. Quain[2], and Katherine A. Smart[1]*

[1]Department of BMS, Oxford Brookes University, Headington, Oxford, UK. [2]Bass Brewers Technical Centre, Burton-On-Trent, UK.
* kasmart@brookes.ac.uk

**INTRODUCTION.** Ageing in yeast is determined by the number of times an individual cell is capable of dividing, termed its replicative capacity. As a consequence of division by budding both mother and daughter cell retain chitinous scar tissue at the site of cytokinesis (1,2). Daughter cells exhibit a frail structure known as the birth scar, while the mother cell displays a more substantial bud scar. The number of bud scars present on the cell surface is therefore directly related to the number of times a cell has divided. It has been hypothesized that scars may be subject to stretching in conjunction with cell expansion (1), however there has been no previous study to support this observation.

**METHOD.** BB11 cells were age synchronised using a method similar to those reported previously (3). Bud scars were stained using FITC conjugated wheat germ agglutinin (WGA). Cells were examined for size and scar details using a using an LSM 410 inverted laser scanning confocal microscope (Carl Zeiss, Germany) with a 488nm argon ion laser for fluorescence imaging and transmission detector for DIC.

**RESULTS.** Cells were observed to increase in volume from $153.6 \pm 90.2\mu m^3$ (virgin cells) to $390.1 \pm 66.0\mu m^3$ (eighth generation cells). In conjunction with mother cell expansion birth and bud scars were observed to increase in size. Birth scars consistently occupied approximately 5% of the cell surface regardless of age, suggesting that expansion occurs solely as a consequence of cell growth. In addition, birth scars were observed to fade and eventually disappear; 75% of eighth generation cells did not display a birth scar. Bud scars were also observed to increase in size ($1.9 \pm 0.2\mu m$ diameter in second generation cells to $2.3 \pm 0.1\mu m$ in eighth division old cells), but did not fade with age. However, scars present on aged individuals were detected to contain breaks within their structure. These breaks were arranged symmetrically in groupings of 2 or 4.

**DISCUSSION.** Barton (1) hypothesised that bud scars produced prior to the maximum size of the cell being attained would be subject to stretching. The increase in size observed for scars can be attributed to the increase in mother cell volume. It is proposed that chitin rings possess breaks in their structure, which only become apparent on expansion of the cell wall. It is possible that these morphological characteristics result purely as a function of somatic growth. However due to their uniform structure, arrangement and positioning around the ring, it is suggested that bud scar breaks are an essential genetically predetermined structural design feature. It is proposed that the opposing position of breaks may function to permit the bud scarring to stretch, despite the inelasticity of chitin, at a constant rate in each direction, thus preventing an uneven distribution of scar tissue and serving to eliminate a potential source of damage to the cell.

**ACKNOWLEDGEMENTS.** This work was funded by a BBSRC grant and supported by Bass Brewers.

**REFERENCES.**

1. Barton, A.A. (1950) J. Gen. Microbiol. 4, 84-86
2. Bacon, J.S.D., Davidson, E.D., Jones, D., and Taylor, I.F. (1966) Biochem. J. 101, 36c-38c
3. Egilmez, N.K., Chen, J.B., and Jazwinski, S.M. (1990) J. Gerontol. 45, B9-B17
